# Extra Supplement—The Nursing Mirror

**Published:** 1890-10-11

**Authors:** 


Being the Extra Nursing Supplement of "The Hospital" Newspaper.

All Contributions for this Supplement should be addressed to the Editor, The Hospital, 140, Strand, London, W.C., and should have the word "Nursing" plainly written in left-hand top corner of the envelope.

### En Passant.

A LETTER TO NURSES.—The eighteenth quarterly letter to the Mary Adelaide nurses is written by Mrs. Fuller, and deals with the Pension Fund. She says: "And now, at the risk of being thought prosy, one word again on the especial subject I have been asked to write about. I want to use *my* influence to persuade you all to join the Royal National Pension Fund; and the sooner you do it the better. You will feel much more comfortable knowing you have a 'stand by' in case of anything happening to you." Some cases are given, one of a nurse, only 30, who would have greatly benefitted, and been saved from the degradation of begging, had they only joined the fund.

THE CANCER HOSPITAL, FULHAM ROAD.—On the invitation of the matron (Miss Rogers), who has been spending her well-earned holiday at Sevenoaks, and her friends, Mrs. Ferguson and Miss Farquharson, the nurses of this hospital have recently had an opportunity of enjoying picnics at that delightfully picturesque place. The weather was all that could be desired, and after a most enjoyable outing, spent in roaming through Lord Sackville's charming house and grounds at Knowle, and in drives about the neighbourhood, the nurses returned to town much delighted with the holiday, and highly appreciating the kindness and hospitality of the matron and her friends.

BATTLE.—This town is to have a local Benefit Association for Providing Cottage Nurses, and a meeting was held last week, with the Dean of Battle in the chair, at which Mrs. Stuart Wortley and Miss Broadwood, the foundress of the original association, spoke. Eleven parishes have combined, which guarantee a yearly subscription of 25s. for every hundred of the population. Miss Broadwood advocated her idea that poor people, who are glad to get good nursing attendance from trustworthy women of their own class who blend good nursing knowledge with help in the duties of the household, are not prepared to avail themselves of the assistance of highly-trained nurses whose services are limited to pure nursing duties. But what "good nursing" is is a much vexed question. We have hitherto thought that thorough training is an absolute necessity to it. Sussex seems, however, to be of a different opinion.

SHORT ITEMS.—Miss Ashby is district nurse at the new Catholic Institution in Ladbroke Road.—A testimonial is being subscribed for for Mrs. Adams, for thirty years matron of Tunbridge Wells Hospital.—A nurses' dormitory, to hold 140 beds, is being added to Edinburgh Royal Infirmary.—Several American ladies are forming themselves into an association to secure pensions for the nurses who worked through the Civil War, many of whom are now in poverty.—In Chicago there are 147 nurses, three colleges of midwifery, and nine training schools for nurses.—Mrs. George Gates has built and furnished a nursing home to Buffalo General Hospital at a cost of £2,800.—The Blue Book of the proceedings before the Select Committee of the House of Lords on Hospitals can now be had, price 5s.—According to a law lately passed in New York, a nurse who notices any inflammation of the eyes in infants under two weeks old must immediately call in medical advice. It is thus hoped to arrest the spread of blindness.

THE FIRST THOUSAND.—Some 500 photographs have been received, and the others should be sent in within the next ten days at latest. They are coming in very fast, and we hope no nurse will voluntarily allow herself to be excluded from this historical group by dilatoriness or indifference.

A TRIBUTE OF PRAISE.—Macclesfield nurses may well be proud of the following letter from the father of a late patient: "Dear Mr. Corbishley,—Will the governors of the infirmary kindly accept, for the institution, the enclosed cheque, as an acknowledgement of my very sincere thanks for the services rendered to my son by their medical officers and staff of nurses, and at the same time allow me to express to them my admiration of the manner in which the institution seems to be conducted. My visits to my son, although mixed with some considerable anxiety, have been of interest and pleasure, which the assiduous care and attention on the part of the nurses gave to me. My impressions have been that (if not of the most important to patients) so devoted and efficient staff of nurses must be of great value to both medical officers and patients, to the former in carrying out their instructions, and to latter's benefit."

LONDON HOSPITAL NURSES.—The article in the *New Review*, by Miss Lückes, tells a wonderful tale of the steady progress in nursing at that hospital since 1880. In the course of ten years the staff of nurses has been doubled; they have been provided with separate bed-rooms and set meals; the daily hours off duty and the annual holiday have been doubled; and everything has been done to make the nurses happy and healthy that the committee felt to be absolutely necessary. There were no ward-maids at the London Hospital ten years ago, and the special nurses were chosen from the scrubber class, and paid 1s. 6d. a-night. With regard to the sleeping room, Miss Lückes says: "The trial to women of the better class, of never being alone for five minutes out of the twenty-four hours, is one that, perhaps, can hardly be estimated without personal experience of it." After stating the successive stages of improvement of the last ten years, Miss Lückes concludes with this opinion: "On a careful review of these plain facts, I find infinitely more grounds to justify the contented appreciation of a large majority of our workers, than reason to complain that more has not already been done for the benefit of the nursing staff." To which words, those of us who know and love the dear old London Hospital, add a hearty, "Them's my sentiments!"

A TRAINED NURSES' AGENCY.—A new movement in Melbourne, Australia, needs special mention. It is the formation of a Trained Nurses' Agency at 58, Russell Street, under Mrs. R. C. Norman. Nurses pay an entrance fee of 5s., and a fee of 2s. on each case secured through the agency. We regret to see there is a system of fines, and that the nurses take no part in the management of the agency, which seems to be a purely private venture. The good points are the formation of a nurses' club, which was opened on September 1st, in connection with the agency, the subscription being a guinea per annum. Light refreshments and light literature are supplied at the club, which is open daily from nine a.m. till ten p.m., and on Sundays from two to nine p.m. Another good point is the publishing of a list of the names and qualifications of the nurses: we observe on the list Alice Baxter, of Manchester Infirmary; Mrs. E. Chequer and Mrs. Mary King, of Guy's; Mrs. O'Flaherty and Mrs. Mina Ould, of St. Thomas's. It seems almost as though marriage was a Melbourne qualification for a nurse! There are also the names of some male nurses on the list.

### Sketches of Insanity.

By Francis H. Walmsley, M.D. (Senior Assistant Medical Officer of Leavesden Asylum).

(*Continued from page* viii, *Vol. VIII.*)

### XIII.—ALCOHOLIC INSANITY.

Alcohol is absorbed directly into the circulation, and is capable of acting as a direct poison upon the nervous tissue. Taken to excess it is, in its passage through the organism, everywhere equally a destroyer. It causes profound changes in every tissue and in every organ of the body, but of all the symptoms of chronic alcoholism those connected with the nervous system are the most characteristic. Indulged in immoderately, alcohol leads either to formidable maniacal excitement or to the pitiable spectacle of acquired dementia.

Dipsomania.—The subjects of this disease are governed by an overpowering morbid impulse to indulge in intoxication at all risks. The habit of drinking, or some inherited or other cause has set up the diseased condition. Chronic habitual drinking is undoubtedly hereditary in many cases; not that the ancestors have necessarily been drunkards, but that the family is of unstable nervous organization. This constitutional infirmity is handed down as a heritage to the offspring, and is manifested in them by an intense craving for drink. The following case is quoted as illustrative of the dipsomaniacal condition: An individual of good social position, of exceedingly active business habits, and one who always lived a sober and circumspect life except when the fit was upon him, did not go far off to avoid scandal, but went to the nearest public-house, and shut himself up in a back parlour with disreputable companions and drank brandy for six weeks—all this close to the village where he was an important man—during the paroxysm being totally regardless of his health, his reputation, and of all that could make life dear to him.

Acute Alcoholism, or Delirium Tremens.—In this disorder the symptoms creep on gradually, the patient loses appetite, takes an aversion to food, becomes restless and wakeful, his sleep—obtained in unrefreshing snatches—is disturbed by horrible dreams, the face is pale, the skin bathed in perspiration; there are twitchings of the hands and trembling of the muscles of the face, limbs, and body generally—the patient can be felt to vibrate, so to speak. The trembling is sometimes so convulsive as to make the step hesitating, and the taking up of objects almost impossible. Fluids cannot be conveyed to the mouth without spilling. The patient is frightened, restless, and agitated; he is in a state of mental perplexity, talks ramblingly and incoherently, his delirium may vary from quiet wandering to wild maniacal excitement, marked by hallucinations and delusional ideas; he displays a painful eagerness to do something on which his mind is bent, wishes to start on a journey to some place—it matters not where—his desire is to get away from himself. His delusions and hallucinations are the outcome of the abnormal information conveyed to his mind by his disordered senses—particularly the sense of sight—thus he fancies himself surrounded by hideous and loathsome objects, such as toads, serpents and reptiles, or that insects, beetles, or mice are creeping over his body, or he is persecuted by strange sounds; suspicious and frightened he searches behind the bed-curtains, under the bed, or in corners to satisfy himself there is nothing hiding there, threatened by assassins, attacked by robbers, he is a prey to a thousand miseries. He looks upon everybody with terror and distrust, imagining they are trying to poison or otherwise injure him. Under the influence of this vague dread, he displays resistance and violence to those about him, and may do grievous bodily harm to himself or others; even in cases in which there is extreme violence marked by sudden explosive impulses, culminating in homicide or suicide, the excitement results from fear—for terror itself has its times of audacity. This is the condition known as alcoholic mania, and it is the physical explanation of many fearful crimes; not unfrequently, when in this state, individuals suddenly assault and kill their friends and associates under the belief that they are evil spirits or strangers who have come to attack them.

Trousseau remarks that the drunkard who has come through an attack of delirium tremens is as seldom cured of his passion for alcohol as is the gambler of his passion for play.

Chronic Alcoholism is marked by general muscular restlessness and unsteadiness, there are twitchings and trembling of the lips and facial muscles, of the fingers, hands, and limbs, the patient is subject to frequent attacks of vertigo, giddiness or faintness; his face is dull, stolid, and expressionless; the habitual immoderate drinker exhales a distinct alcoholic and saccharine odour—peculiarly unpleasant.

There is advancing and persistent mental enfeeblement, indicated by loss of energy and will-power, and lack of judgment; notwithstanding this deterioration, however, it is curious to observe that the individual frequently entertains elevated ideas of his own worth and importance—ideas akin to the delusions of grandeur seen in other grave forms of insanity—he manifests serene contentment and satisfaction with himself and his surroundings.

The decadence of the moral sensibility follows that of the intelligence step by step, the whole man—body and mind—is gradually but certainly transformed; he becomes less scrupulous, less conscientious, he ceases to be exact in his transactions, he grows vacillating and unreliable, it is impossible to put any dependence on his promises, in his relations with his fellows he too frequently betrays selfishness untruthfulness, cunning, and deceit. His power of self-restraint is manifestly weakened; from slight causes he is readily moved to increasing irritability and uncontrollable outbursts of temper. His mind is filled with suspicion and distrust, his imaginary evils soon pass into real ones; delusions are developed which tend to become fixed and persistent; thus, he believes that electricity or mysterious agencies are at work to injure or torture him, that attempts are made to poison him, his enemies are at hand plotting his destruction; he is tormented by voices prompting him to commit hideous crimes. Under the influence of these delusional ideas, he is frequently driven to acts of desperate violence.

This tendency to suicidal and homicidal impulses is a most prominent feature in both the acute and the chronic forms of alcoholic insanity, and should ever be borne in mind by those having charge of such patients.

As the disease progresses the memory undergoes further disorganisation, or is altogether annulled, the patient forgets everything of the recent past and most of what happened in his previous life.

In very advanced cases of alcoholism the individual becomes indifferent, apathetic, and stupified, he is lost to all healthy feeling and human interests; all intelligence is abolished, the mind becoming a perfect blank.

Physically there is complete prostration of muscular strength, the limbs are wasted to the last degree, the arms are so paralysed that the patient is totally unable to do anything for himself, there may be a wide extent of numbness or tingling, the sensibility of the skin is blunted, when pricked the patient does not flinch; owing to failure of the muscular sense he is unable to obtain a correct view of the situation of different parts of the body, with eyes shut he cannot tell the position of his legs—cannot cross one over the other—similarly he is unable to place his index finger upon the tip of his nose; the sight is weakened, the pupils less sensitive, a feather may be passed over the eye without causing pain or movement, objects appear tremulous and transient, blindness is not uncommon; there may be deafness and loss of the sense of smell and of taste.

Finally the patient may be seized with an epileptiform convulsion ending in coma and death. To this pitiable condition does the man, enslaved by his passion for drink, reduce himself! Half the misery and wretchedness of human life, and much of the crime of the land is but the outgrowth of a state of mind and morals produced by drink.

(*To be continued.*)

### Notes from Australia.

(*By Our Own Correspondent.*)

OUR late Governor, Sir Henry Loch, and Lady Loch, are, I hear, pursuing their benevolent work at Cape Town, Africa. The Sisters do extremely good work in the hospital there, but some reforms in management are needed, and to these Lady Loch has turned her attention. She has both tact and kindness, as the nurses of Melbourne know well.

There is little to tell of the Charities Commission; it has been diverted to the condition of the Melbourne Hospital building, just as your Lords' Committee has had its attention diverted to the nursing of the London Hospital. The similarity between the two Commissions is most amusing; the governors of the Melbourne Hospital are most indignant at the charges brought against it; they acknowledge that the hospital wants re-building, but ask is it their fault that they have no funds? They also feel that Mr. Fergusson had no right to bring the charges forward in such a public and such a sensational form. Really the secretary of the London Hospital and the secretary of the Melbourne Hospital might exchange condolences. The annual meeting of the Melbourne Hospital was held on July 29th; the report stated that the receipts for the year for the purposes of maintenance, inclusive of £14,000 allocated to the hospital from the vote of the Legislature, £2,985 4s. 2d. from the Committee of the Hospital Sunday Fund, and £7,479 18s. 1d. from subscriptions and sundry sources, make a total revenue of £24,465 2s. 3d. On the other hand, the expenditure for the same purpose has amounted to £29,427 18s. 8d., leaving a balance to debit of £5,021 0s. 3d. During the period embraced in the report medical and surgical assistance has been given to 22,394 cases; of this number 18,161 have been treated as out-patients, 4,223 have been admitted to the wards. Of these 3,098 have been discharged, cured or relieved, 103 have left as incurable or for various reasons, 762 have died, and 270 remained in the institution on the 30th June. Of the number of fatal cases 241 died within 72 hours of their admission. Without any desire to anticipate recommendations which may be decided upon, the Committee view with satisfaction the appointment of the Royal Commission on Charitable Institutions. In this relation the Committee would remark that they have always recognised the defects of construction in the older portions of the hospital buildings, and will be prepared to adopt recommendations for the erection of a hospital commensurate with the importance of the city, provided the necessary funds are forthcoming.

The annual meeting of the Alfred Hospital was held on July 25th, and the report referred specially to the nursing department. Twenty-five pupils had entered the training school during the year, 18 left after obtaining their certificates, six resigned, two were dismissed, and one died, leaving 24 pupil nurses still in the institution. Only three students, all of whom were ladies, entered the clinical school for medical practice during the year, but it was anticipated that the number would be largely augmented when the hospital extension had progressed far enough to allow of suitable accommodation being afforded the students. One hundred and ninety-six patients had been treated in the paying wards, of whom 151 were discharged cured or relieved, 36 died, and nine remain in the institution. In the general wards 1,612 patients had been treated, and the outdoor patients had numbered 2,267.

The Countess of Hopetoun has opened the new home of the Convalescent Aid Society; the home will accommodate twenty-five inmates. Mrs. Gibson is the lady superintendent. The Homœopathic Hospital has commenced its course of winter lectures for nurses; the training school there is prospering. The great want in Melbourne is more practical knowledge on the part of committees; ladies and gentlemen of wealth think they perform their duties to their poorer brethren if they sit on one or two committees of charitable institutions, but they do not give sufficient time and consideration to study the internal working of these charities. If every lady governor of a hospital had spent three months as a pupil nurse within its walls, they would be more ready to consider the needs of nurses, and not regard them as so many troublesome domestic servants.

A terrible case of diphtheria attacking a whole family has occurred. First, the eldest girl, aged six years, was attacked and died within two days; a week after the eldest boy died, then the second boy; and the mother, aunt, two professional nurses, and the sole remaining child are all seriously ill, and four of them have been removed to the hospital. One nurse refused to go to the hospital.

It is impossible to conclude an Australian letter just now without one word anent the strikes and the unemployed. The latest bulletin from the Salvation Army says that 170 men are wanted for labour work, and only 72 have applied; so it is not a case of starvation. This is where the evil lies —the men want work in town; they won't go into the bush. They are social fellows, and a lonely life is abhorrent to them. So they are going to try and force employment in the towns, which is, of course, an impossibility for all. Your poor in England are crying for land—here is land in plenty to be had for the asking, but the men won't go and work it.

### Death in Our Ranks.

THE death is announced of Mrs. Mackay, of Golspie, who was one of the first nurses enlisted by Miss Florence Nightingale for service in the Crimea. Mrs. Mackay was a native of Strathy, in the north of Sutherlandshire, and on the outbreak of the Crimean War accompanied her husband, Sergeant Mackay, of the 42nd Highlanders, to the East. She remained in the Crimea during the whole of the war, and served under Miss Nightingale with great skill and unremitting tenderness. There was a large public gathering at her funeral, at which her aged husband, a brave Crimean veteran, was present.

THE death is also announced of Mrs. Hampson, well known in Manchester as Caroline Passman. She studied at one time at St. Mary's Hospital, Manchester, as a monthly nurse, and she worked hard to secure the repeal of the Contagious Diseases Acts. She died at a home, at Highbury, which she herself had founded. Her whole life was one long labour to ease those sick in mind and body.

ON October 6th at the London Hospital, Nurse Annie Aris, aged 33, from typhoid fever, contracted while nursing a private patient away from the hospital.

"Tis finished; all is finished,
Their fight with death and sin;
Fling open wide the golden gates,
And let the victors in."—*Dean Alford.*

### Presentation.

MISS RENNIE, late Sister Dorcas of the Aberdeen Infirmary, has been presented with a handsome lady's companion by the nursing staff. Miss Rennie is leaving to start a private pay hospital in Aberdeen. She holds the Guy's certificate, and has had much experience in nursing work. We hope her venture will be successful.

### Lectures and Amusements.

INSTITUTE OF MEDICAL ELECTRICITY, 35, Fitzroy-square.—A course of ten weekly lectures on Electro-Therapeutics commenced on October 7th at eight p.m., and will be continued on subsequent Tuesdays. A course of nine lectures on Masso-Therapeutics will commence on October 10th at eight p.m. Lecturers: Dr. Harries and Mr. Lawrence. Fee for nurses, one guinea each course.

NURSES' CLUB, 12, Buckingham Street, Strand. — On October 10th Dr. W. S. A. Griffith will lecture at a quarter to eight.

ST. MARYLEBONE PARISH CHURCH.—The oratorio of "St. Paul" will be given at eight o'clock on October 16th, 23rd, and 30th.

WE would also remind our readers that the popular concerts at St. James's Hall commence on the 20th, and the Crystal Palace concerts on the 11th.

### Everybody's Opinion.

### THE NURSES OF THE LONDON HOSPITAL.

"MRS. M." writes: It seems as though no amount of positive truth will convince the "agitators" that their charges against the management of the London Hospital, when not absolutely untrue, are grossly exaggerated.

I think I may claim to have some experience of the nursing arrangements at the London—as I was Sister there for some years—and to me it seems a great misfortune that, through ignorance, so much harm is being done to nurses by those who profess to be the nurses' friends.

There is an accurate list of the non-nursing work done by the nurses at the London Hospital, given in the *Pall Mall Gazette* of the 25th inst., and it really sounds quite alarming.

One great grievance seems to be that the nurses clean the lamps! People reading this might well imagine that the whole of the building was lighted entirely by lamps that were cleaned by the nurses. What is the fact? Why, that each ward is provided with one small lamp, which is used for surgical examinations. Now I believe most people will agree with me that there is nothing of what is supposed, in private houses, to be a servant's duty, that we have so much difficulty in getting properly done as the trimming of the lamps. How often the annoyance of sending the lamps to be retrimmed, I am sure, is recognised by all who do not trim them themselves.

When I was Sister I very often trimmed the lamp myself. We must remember how very necessary it is that they shall be perfectly bright and smokeless. I can remember the late Mr. James Adams once examining a patient's eyes in one of my wards, and turning to me, saying, "That lamp is properly trimmed, Sister. I suppose you did it yourself;" and then saying how strange it was that lamp trimming was one of the things servants never did properly.

I am perfectly sure no Sister would trust the cleaning of the lamp to the wardmaid.

Much stress is laid on the fact that the nurses clean the lavatories. A most necessary piece of nursing work, I consider. All the excreta for examination are kept in the ward lavatories, and the most casual reader will immediately understand how important a part of a nurse's education the disinfecting of the utensils, and the keeping numbered, and in their right places, the vessels containing the matter for examination is. The actual cleaning of the lavatory is done by the wardmaid.

How can any outsider understand all this sort of thing? And if a probationer having failed in her attempts at nursing gives as a reason to her friends that she was overworked, and as an example of that overwork, that she had to "clean the lavatories," what wrong ideas the public must get of a nurse's work! The only thing quoted that is not a nurse's work is the attending to the Sister's room, and even that is greatly exaggerated.

I, when sister, always made my own bed, and the wardmaid did my room. Certainly, the nurse brought my breakfast to me. Perhaps I was particularly fortunate, but I know all the nurses that ever worked in my wards were only too pleased to do anything of that kind for me without being asked to do so. Another gross misstatement is that these probationers were held solely responsible for the nursing of the patients in the wards in which they worked. The Sister in charge of the wards, whether day or night, is responsible to the matron for the nursing of each patient. The employment of women of a better social standing than the old style of hospital nurse is of great service by reason of the refinement brought into the work, but the good thus gained is in danger of being done away with, for after such an attack being made on a training school such as that of the London Hospital, where the nurses are shown every consideration, superintendents will be very chary of engaging any woman whose social position is superior to that of the old style of nurse. There was a suggestion made at the inquiry at the House of Lords that the twenty-four hours' work should be divided into three—that is to say having three changes of nurses during the twenty-four hours. I think the patients (whose welfare seems to be considered in these complaints very far behind the nurses) would object very strongly, and I am quite sure it would be bad for their recovery. Many patients object even to the alternate day and night nurse (especially in private or special cases). Then the immense amount of alteration and re-arrangement in any hospital would be appalling, besides the extra expense. I believe the matron of any hospital would welcome more nurses, if funds would allow it, and I am sure Miss Lückes who, has worked so hard and who has so patiently fought each obstacle in her path until the training school at the London is second to none, would be one of the first to gratefully acknowledge such public generosity that would enable the committee to build another nursing home.

"D. C." writes: Rumour says that, with the exception of two nurses and perhaps a single sister, the whole of those who work under the matron at the London Hospital are thoroughly contented and happy and loyal. Anyone who takes the trouble to go through the wards will see at a glance how absurd are the reports that depict the nurses as worn out and broken down with a "leisureless toil," and other fearful and inhuman atrocities! It is to be hoped that those of the public who are sufficiently interested will go and put the question to this, the most practical proof.

"MEDICUS" writes: I am sure our nurses would be less healthy if the sentimental public gained its point and stuffed them with pastry and made dishes. Plain, wholesome work demands plain, wholesome food; for myself, I never eat meat more than once a day, and we doctors have for years persistently tried to impress on the general public that most of its illnesses arise from overfeeding.

### Notes and Queries.

**Answers.**

*Probationers.*—All ladies desiring to know the terms under which probationers are received for training at the different hospitals are requested to see the *Hospital Annual.*

*Nurse Foster.*—Nurses' veiling can be obtained from Messrs. Wallis, of High Holborn, who make a specialité of items of nursing uniform.

*Nurse Wharton.*—Miss Hooper's address is 9, Upper Baker Street, N.W.; you had better write to her. Regarding massage, write to Mrs. Creighton Hale, 46, Maddox Street, W.

*Eliza McB.*—Playfair's "Midwifery," or Barnes' "Manual."

*Hare.*—"The Nightingale" is published at 13 West 42nd Street, New York. English subscribers have it sent by post.

### Vacancies.

**THE following vacancies are announced:**

**Nurses.**

Aberdeen Institute.

Belgrave Hospital for Children, S.W.

Bedford Infirmary (night); £22.

Borough Hospital, Birkenhead; £25.

Bath Home.

Blackheath and Richmond Institutions.

Brixton Institute.

Bromley Institution.

Bournemouth Institute; £25.

Cheltenham Institution.

Cottage Hospital, Epsom; £20.

Cambridge Home.

Easter Ross Poor House; £30.

General Nursing Institute, Covent Garden.

Hanover Institute, W.; £25.

Hull Home.

Homœopathic Hospital, Birmingham.

Halifax Infirmary (assistant); £18.

Industrial School, Swinton, £23.

Jersey Institute, £25.

Jessop Hospital, Sheffield; £25.

King's Lynn Hospital; £20.

Minster Workhouse Infirmary; £20.

Manchester Ship Canal Hospital; £25.

Miller Hospital, Greenwich; £22.

Northwood Fever Hospital (Asst.); £28.

Newark Hospital (Assist.); £18.

Nursing Institution, Wimpole Street, W.

Norwich Home.

Nottingham and Notts Institution; £25.

Ophthalmic School, Hanley; £23 (also Assist., £18).

Royal Infirmary, Bristol; £26.

Rochdale Infirmary; £22.

Rural Nursing Association.

South Devon Hospital, Plymouth; £25.

St. Leonard's, Shoreditch; £20 (also Assist., £17).

St. William's Hospital Rochester; £25.

Southport Infirmary; £25.

Sanatorium, Margate; £24.

St. Saviour's, East Dulwich; £20.

St. Helena Home, N.W.

Sheffield Home; £25.

Sanatorium, Hull; £30.

Worcester General Infirmary; £25.

Whitechapel Infirmary, E.; £20.

West Cornwall Infirmary (Assist.); £16.

Workhouse Infirmary Nursing Association.

Western General Dispensary, N.W.; £25.

Victoria Home, Bournemouth.

Victoria Hospital, Burnley; £26.

**District Nurse.**

Bredbury and Romily Association; £60.

**Probationers.**

Bolton Infirmary.

Horton Infirmary, Banbury.

Leeds Institution; £14.

N.O.C. Hospital, Wisbech.

Monsall Fever Hospital; £15.

Miller Hospital, Greenwich.

St. Helena Home, N.W.

**Sisters.**

South Devon Hospital, Plymouth; £30.

Swansea Hospital; £25.

### Amusements and Relaxation.

**N.B.-THIRD QUARTERLY WORD COMPETITION**

**Commenced Oct. 4, 1890; ends Dec. 27, 1890.**

The word for dissection for this. the SECOND week of the quarter, being "CHELSEA."

| Names. | Oct. 2nd. | Totals. | Names. | Oct. 2nd. | Totals. |
|---|---|---|---|---|---|
| Dulcamara | — | 508 | Jenny Wren | 119 | 885 |
| Tinie | 134 | 964 | Rhoda | — | 242 |
| Henri | — | 395 | Annabel | — | 17 |
| Agamemnon | 132 | 982 | Spare Moments | — | 19 |
| Patience | 132 | 964 | Qu'appelle | 116 | 917 |
| Brisbane | — | 332 | Nurse | — | 29 |
| Nurse Hilda | 111 | 883 | A. B. C. | — | 251 |
| Lightowlers | 124 | 942 | Brickbat | — | 77 |

**Results of Second Quarterly Word Competition.**

*First Prize*, 15s., is awarded to Agamemnon (Mr. H. Hull, Weybridge, Surrey). *Second Prize*, 10s., a tie between Patience (Miss Calisè, Newbury) and Tinie (Miss H. Todd, Craven Villa, Newbury); we therefore give them the choice of dividing the prize equally, or of having another word set to decide the tie. *Third Prize*, 5s., is awarded to Lightowlers (Miss Chadwick, 2, Canning Street, Liverpool).

